# Supplementary material for: How structured cultural changes can reduce cesarean section rate in a Danish tertiary hospital
Source: PLoS One. 2025 Nov 17;20(11):e0336474. doi: 10.1371/journal.pone.0336474 (PMC12622832; doi:10.1371/journal.pone.0336474)

SUPPLEMENTARY 1

# The stepwise initiative

This document is a detailed description of the stepwise introduction of twelve initiatives to reduce CS. For further information, Kamilla Groenemeijer Nielsen, co-author, may be contacted: kamillagnielsen@dadlnet.dk. The following are placed in chronological order according to the implementation.

| **The stepwise initiative to reduce cesarean section** |
| --- |
| \|  \| **The 12 initiatives** \| **2008** \| **2009** \| **2010** \| **2011** \| **2012** \| **2013** \| **2014** \| **2015** \| **2016** \| **2017** \| \| --- \| --- \| --- \| --- \| --- \| --- \| --- \| --- \| --- \| --- \| --- \| --- \| \| 1 \| Monitoring obstetric results \| **x** \| **x** \| **x** \| **x** \| **x** \| **x** \| **x** \| **x** \| **x** \| **x** \| \| 2 \| Birth planning Clinic \| **x** \| **x** \| **x** \| **x** \| **x** \| **x** \| **x** \| **x** \| **x** \| **x** \| \| 3 \| Postpartum consultation \|  \| **x** \| **x** \| **x** \| **x** \| **x** \| **x** \| **x** \| **x** \| **x** \| \| 4 \| Supervision \|  \|  \|  \| **x** \| **x** \| **x** \| **x** \| **x** \| **x** \| **x** \| \| 5 \| Proactive approach \|  \|  \|  \|  \| **x** \| **x** \| **x** \| **x** \| **x** \| **x** \| \| 6 \| Morning conference \|  \|  \|  \|  \| **x** \| **x** \| **x** \| **x** \| **x** \| **x** \| \| 7 \| Education & training \|  \|  \|  \|  \| **x** \| **x** \| **x** \| **x** \| **x** \| **x** \| \| 8 \| Improved homestays \|  \|  \|  \|  \| **x** \| **x** \| **x** \| **x** \| **x** \| **x** \| \| 9 \| Consensus decisions \|  \|  \|  \|  \| **x** \| **x** \| **x** \| **x** \| **x** \| **x** \| \| 10 \| Public involvement \|  \|  \|  \|  \|  \|  \| **x** \| **x** \| **x** \| **x** \| \| 11 \| Midwife coordinator \|  \|  \|  \|  \|  \|  \| **x** \| **x** \| **x** \| **x** \| \| 12 \| Teamwork \|  \|  \|  \|  \|  \|  \|  \|  \| **x** \| **x** \| |

**1. Monitoring obstetric results**

Data on the mode of delivery, inductions, CSs, vacuum deliveries, episiotomies OASIS, postpartum bleedings, Apgar scores and umbilical cord pH in all Robson groups were gathered daily and evaluated monthly with doctors, midwives, and midwife-coordinators. The data provided the basis for continuous adjustment of clinical practice, always striving to reduce interventions while keeping complications at a low level.

**2. Birth Planning Clinic**

A Birth planning clinic was introduced for couples requesting a planned CS. If the woman/the couple wished for a CS without a medical indication their reasons were respectfully discussed and met with empathy. Any previous births were reviewed from hospital records, including the partogram and CTG trace, always with respect for the couple´s experience. At the end of one or more meetings with a doctor or a midwife from the birth plan team, a personal birth plan was made. The couple had to feel safe with the birth plan to go on to planning a vaginal birth. This included timeouts during labour anytime events in labour were not meaningful to the couple. This timeout would be held with the obstetric and midwifery team on call. In a timeout, the distressing issues were evaluated and solved, and a specific plan was made that the couple agreed to. Part of the birth plan was a visual birth diagram that showed the specific steps of labour and an expected timeframe. This diagram was used to create an overview and to identify what specific parts of the birth triggered fear (the birth diagram is shown at the bottom of this supplementary). The birth plan was an integrated part of the woman’s hospital record, and all staff was dedicated to following it. If the woman maintained her wish for a CS after the consultations, the request was brought to the doctors and midwife conference.

**3. Postpartum consultation**

All couples were offered a midwifery consultation two days after delivery, helping them verbalise the family's experience of childbirth. If needed, a further follow-up consultation was planned one month later to sum up the process.

All women who had experienced an emergency CS or other emergencies, met the doctor in charge of the procedure before they left the hospital. The intention was to clarify the reasons for the emergency, to answer any questions the couple might have, and to make a recommendation on the mode of delivery for a potential next birth.

**4. Supervision**

An overall approach was, that if the staff felt safe in their work environment, the patients would feel the same. Coaching groups for midwives met 2 hours every 3 months and were led by coach-educated midwives in the unit. The birth planning team received supervision from trained psychologists on difficult cases.

**5. Proactive approach**

An obstetrician went through all antenatal records with documented risk factors. Women with previous CS, previous traumatic childbirth, previous severe postpartum haemorrhage, vacuum delivery, or a wish for CS, were offered a consultation with an obstetrician, in addition to the first-trimester ultrasound scan.

**6. Morning conference**

The morning conference was the centre of all decisions striving for a common culture in the unit with regards to shift change at the delivery suite, obstetric emergency clinic, postnatal ward and gynaecological unit. This gave daily time to go through interventions of the last 24 hours in the delivery suite, led by the midwifery coordinator. The interventions were assessed with respect for the staff that had made the decisions. All knowledge was regarded in the light of learning to discuss possible alternative actions and whether the intervention might have been avoided.

**7. Education and training**

Midwives and assistants from the delivery suite had a full-day meeting and teaching of new and existing procedures 4 times a year. Doctors and midwife coordinators had weekly formalised teaching. The unit annually gathered all staff to do neonatal audits on all cases with a pH < 7.00 and Apgar <7 at 5 minutes.

All new staff was certified in CTG and STAN interpretation with daily training at the morning conference. Yearly team training in obstetric emergencies for all staff and the anaesthetic unit was offered. Upright breech birth has been trained regularly since 2017.

**8. Improved homestays**

All pregnant women with complications were treated according to the guidelines, but unless there was an absolute indication, the woman stayed in her own home. Outpatient controls and the opportunity to contact the unit anytime needed were offered to all.

**9. Consensus decisions**

If a woman wished for a CS or induction without medical indication the case was discussed at a conference with doctors and midwife coordinators to discuss alternative solutions, for example, early induction of labour. Any wish for induction of labour without medical reason, was also decided after consensus. If a woman had taken part in the birth planning but still did not feel safe enough to go through with vaginal birth, an elective CS was offered.

**10. Public Involvement**

The unit shared a common belief; that vaginal birth is better than CS, if safely performed with a good birthing experience for the woman. The aim to help women to a safe and good birthing experience was shared in meetings with the general practitioners and publicly with the people living in Southern Jutland (e.g. articles in local newspapers)

**11. Midwife coordinator**

A midwife coordinator was on call for 24-hour shifts to have a continuing overview of all ongoing births. They were available for second opinions to obstetricians and midwives in all births and were part of the decision-making team in case of interventions. They acted as a buffer when the unit was busy.

**12. Teamwork**

The teamwork between doctors, midwife coordinators and midwives ensured that involved professionals were joined when making difficult decisions or handling emergencies. During labour, obstetricians, midwives, and midwife coordinators were considered a team.


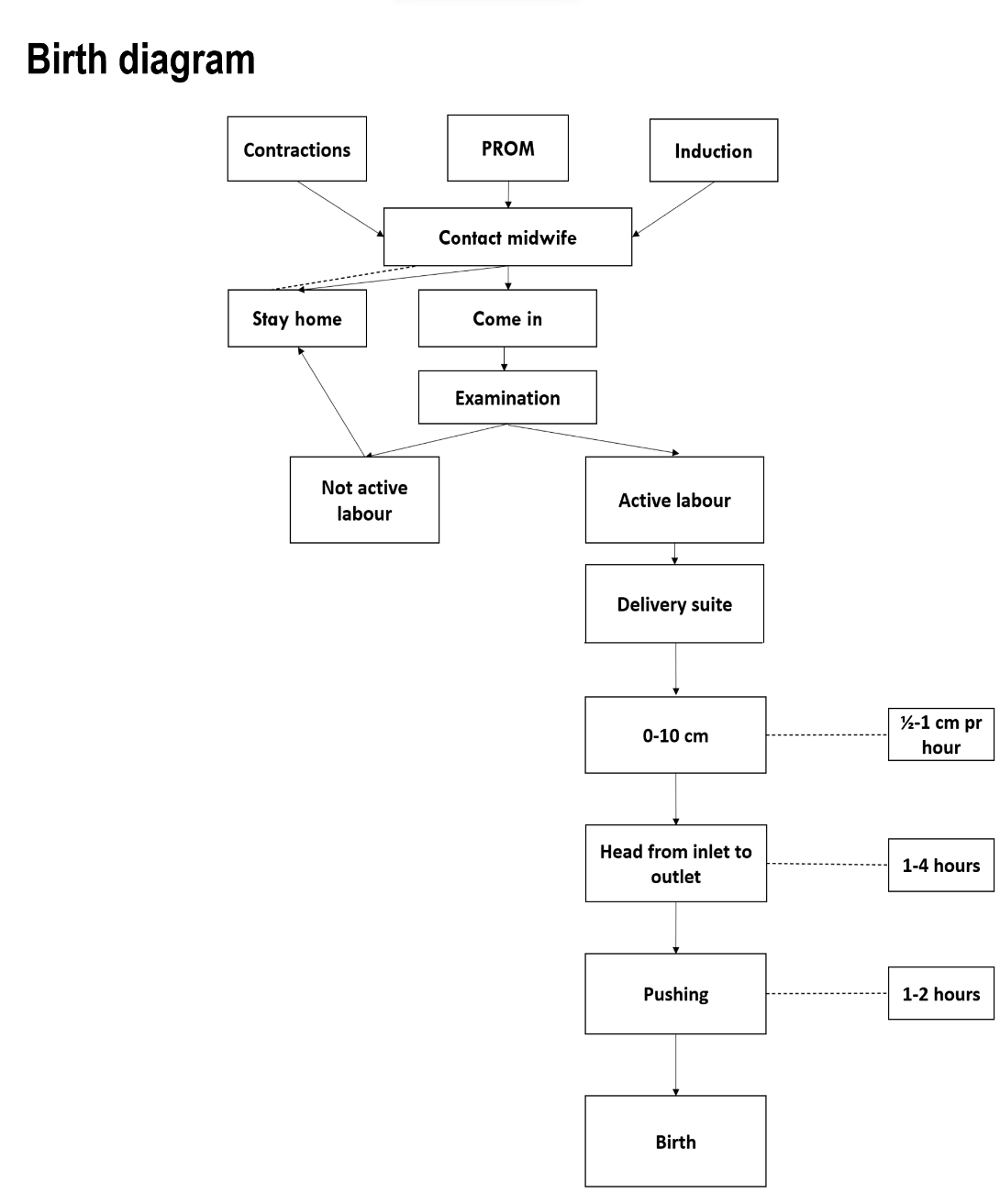

Supplement: S1 File — (DOCX) [file pone.0336474.s001.docx]
