# Supplementary material for: How structured cultural changes can reduce cesarean section rate in a Danish tertiary hospital
Source: PLoS One. 2025 Nov 17;20(11):e0336474. doi: 10.1371/journal.pone.0336474 (PMC12622832; doi:10.1371/journal.pone.0336474)
Supplement: S1 Table — (RTF) [file pone.0336474.s002.rtf]

SUPPLEMENTARY 2
Absolute numbers among Cesarean Section groups.


	HSJ, n=21,232	Control, n=46,417	Denmark, except HSJ, n=802,177	
	
Total, n	Cesarean, n (%)	
Total, n	Cesarean, n (%)	
Total, n	Cesarean, n (%)	
2003	1427	282 (19.8)	3521	635 (18.0)	56,543	10,193 (18.0)	
2004	1491	260 (17.4)	3956	701 (17.7)	56,238	10,800 (19.2)	
2005	1442	269 (18.7)	4085	754 (18.5)	56,233	10,615 (18.9)	
2006	1404	275 (19.6)	3880	798 (20.6)	56,892	11,134 (19.6)	
2007	1364	275 (20.2)	3773	757 (20.1)	55,994	11,338 (20.2)	
2008	1301	275 (21.1)	3659	831 (22.7)	56,690	11,268 (19.9)	
2009	1257	217 (17.3)	2689	621 (23.1)	54,745	11,271 (20.6)	
2010	1389	259 (18.6)	2811	691 (24.6)	55,136	10,986 (19.9)	
2011	1199	214 (17.8)	2784	615 (22.1)	51,133	10,062 (19.7)	
2012	1252	225 (18.0)	2690	614 (22.8)	50,052	9,852 (19.7)	
2013	1423	211 (14.8)	2434	562 (23.1)	48,290	10,081 (20.9)	
2014	1449	238 (16.4)	2453	564 (23.0)	48,993	9,684 (19.8)	
2015	1484	190 (12.8)	2542	564 (22.2)	50,163	9,540 (19.0)	
2016	1704	233 (13.7)	2571	591 (23.0)	52,695	9,601 (18.2)	
2017	1645	197 (12.0)	2569	555 (21.6)	52,380	9,594 (18.3)	
