# Supplementary material for: How structured cultural changes can reduce cesarean section rate in a Danish tertiary hospital
Source: PLoS One. 2025 Nov 17;20(11):e0336474. doi: 10.1371/journal.pone.0336474 (PMC12622832; doi:10.1371/journal.pone.0336474)
Supplement: S2 Fig — (DOCX) [file pone.0336474.s004.docx]

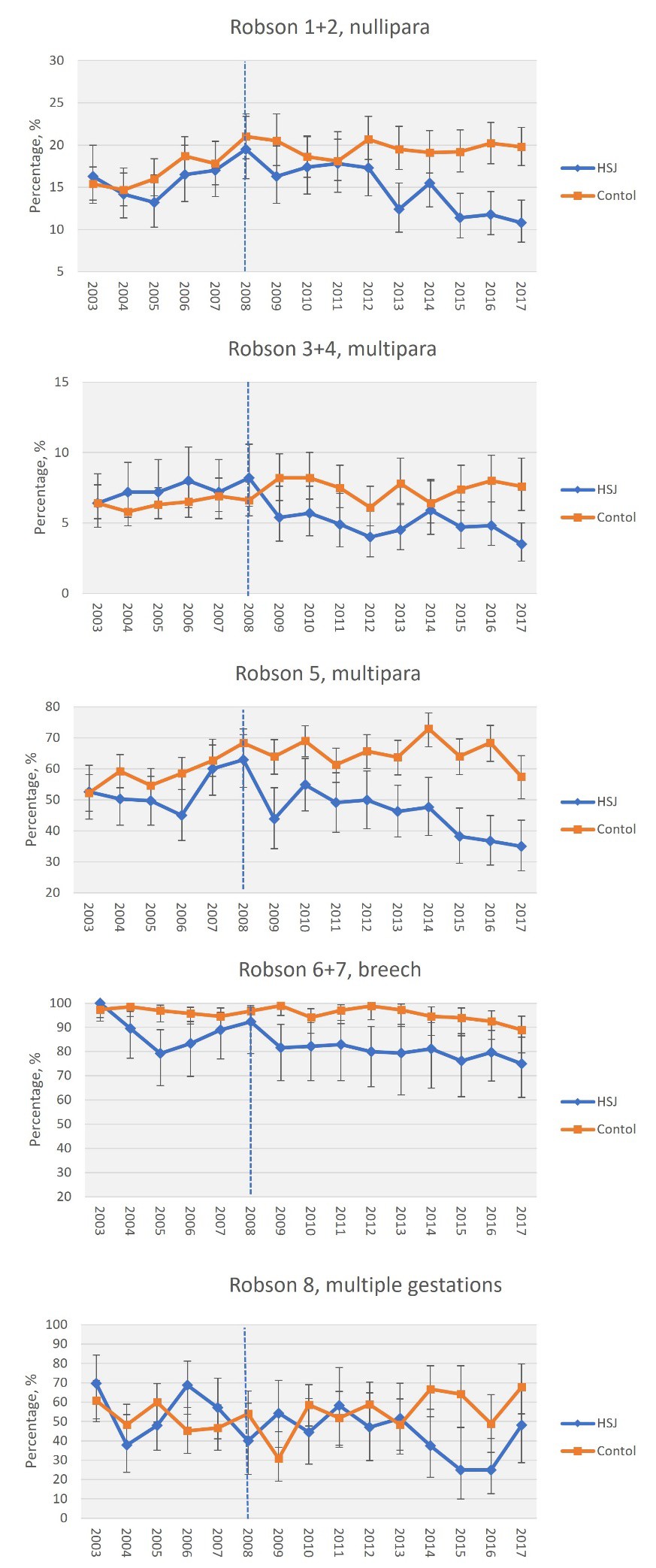


SUPPLEMENTARY 4

Cesarean section according to modified Robsons Ten Group Classification System.

Index hospital (HSJ) and controls. Per cent (%) and 95% CI-interval.
