# Supplementary material for: How structured cultural changes can reduce cesarean section rate in a Danish tertiary hospital
Source: PLoS One. 2025 Nov 17;20(11):e0336474. doi: 10.1371/journal.pone.0336474 (PMC12622832; doi:10.1371/journal.pone.0336474)
Supplement: S3 Fig — (RTF) [file pone.0336474.s005.rtf]

SUPPLEMENTARY 5

Change in maternal characteristics during 2003-2017 among HSJ and control
