# Supplementary material for: How structured cultural changes can reduce cesarean section rate in a Danish tertiary hospital
Source: PLoS One. 2025 Nov 17;20(11):e0336474. doi: 10.1371/journal.pone.0336474 (PMC12622832; doi:10.1371/journal.pone.0336474)
Supplement: Attachment — Submitted filename: Manuscript 251124_ edited by SRD.docx [file pone.0336474.s007.docx]

# Title page:

## How structured cultural changes can reduce caesarean section rate in a Danish tertiary hospital; a controlled interrupted time series analysis.

Eva Rydahl, ^1^; Kamilla G Nielsen^2^; Ole Olsen^3^; Amalie L Henningsen^4^; Helle Johnsen^5^

^1^ Midwife, MHS, Ph.d, Department of Midwifery, University College Copenhagen, Denmark.

^2^ MD, Hospital Sønderjylland, Aabenraa, Denmark

^3^ Statistician, Senior Researcher, University of Copenhagen, Copenhagen, Denmark

^4^ Midwife, Stud. MSc in Midwifery, University of Southern Denmark, Odense, Denmark

^5^ Midwife, MMS, Ph.d, Department of Midwifery, University College Copenhagen, Denmark.

Corresponding author: (ER) Sigurdsgade 26, 2200 Copenhagen N, Denmark. E-mail: [evry@kp.dk](mailto:evry@kp.dk). Phone: +45 31661068

ABSTRACT

# Keywords

Caesarean Section [MeSH], Medicalization [MeSH], Overdiagnosis [MeSH], Vaginal Birth after Caesarean [MeSH], Medical Overuse [MeSH]

### Strengths and limitations of this study

- Based on national registry-based data (2003-2017).
- Changes in clinical practice are analysed through a quasi-experimental design.
- 5 years before, and 10 years during the intervention’s implementation.
- The impact of complex caesarean section interventions is discussed.
- A principal study limitation is the use of observational data.

### Introduction

Globally caesarean section (CS) rates are rising, and projections by the World Health Organization (WHO) suggest that 28.5% of births will be by CS by 2030 [1]. The CS can be a lifesaving intervention, while rates below 10% are associated with insufficient care [2]. Nevertheless, CS rates beyond a certain threshold should raise concerns about the potential iatrogenic consequences for the health of mothers, their off-springs, and future pregnancies [3]. Increasing CS rates have not been accompanied by significant maternal and perinatal benefits, and in 2015 the WHO stated that at a populational level, CS-rates above 10% do not lead to reductions in maternal and new-born mortality rates [3].

An increase in the proportion of nulliparous women and older or more obese women may be associated with the rise in CS rates [4]. Still, these changes are unlikely to solely explain the accelerating CS rates and the variations observed between countries [4]. Among the 34 members of The Organization for Economic Cooperation and Development (OECD), the CS rate varies from 15% to 53% (2019)[5], suggesting considerable variation in otherwise comparable countries. OECD finds these variations attributed to various factors, such as CS availability, obstetric training, financial incentives, and liability concerns. Further, preferences among women may contribute. However, these preferences are likely linked to the organisation of the maternity care system and cultural attitudes towards labour and birth [5].

High rates of CS cause concern as the surgery is associated with both short- and long-term risks. Maternal adverse effects may be organ injury, infection, anaesthesia complications, and thromboembolic disease. In subsequent pregnancies, the risk of uterine rupture, placenta accrete, -previa, ectopic pregnancy, infertility, hysterectomy, and intraabdominal adhesions increases [6–11]. Further, CS may lead to adverse birth experiences and promote poorer parenting behaviours [12]. The newborn has an increased risk of neonatal respiratory distress, asthma and obesity [8,9,11].

Various strategies – both clinical and non-clinical – have been suggested to reduce CS rates [13]. In 2014, The American College of Obstetricians and Gynaecologists published a program to prevent nulliparous women from having a CS [14]. Despite this national initiative, the CS rate has continued to increase in the US and is currently at 32,1% (in 2021) [15]. More recent studies suggest a multi-component approach, which includes factors such as health system organisation, culture, and finance to develop strategies for reducing CS rates [13,16]. Likewise, WHO has proposed a broad approach targeting the birthing population, the health care providers, and the organisation, including childbirth and psychoeducation, implementing evidence-based clinical guidelines and a mandatory second opinion before performing any CS. The use of a collaborative midwifery-obstetrician model of care is recommended [4]. However, despite numerous initiatives aimed at reducing CS rates, the effectiveness of such interventions has been limited so far [16].

*The CS reduction initiative in Denmark*

Denmark is a high-income country with free healthcare access. The primary caregivers during labor and delivery are midwives, and obstetricians are in charge if complications occur. The CS rate is relatively stable at approximately 20%, fluctuating between 19% and 22% [17]. Although low in international comparison, the rates are considerably higher than the ideal 10-15% rate suggested by WHO[3]. In 2008, an urban tertiary hospital in the Southern Region of Denmark, Hospital Southern Jutland (HSJ), introduced a systematic strategy to lower the CS rate without compromising maternal and neonatal health. The motivator for the initiative was an observed increase in the CS rate. A department manager inspired by in-hospital birth clinics in Sweden was engaged to support the cultural and structural changes [18]. The Swedish clinics were known for high maternal satisfaction, personalised care, informed decision-making, and collaborative patient-provider communication [18].

The initiative at HJS included cultural, structural, and clinical changes. First, common values were agreed upon between midwives, obstetricians and other staff; that vaginal birth and a good birthing experience are important, that obstetric knowledge and knowhow are essential when safely reducing intervention rates, and that staff who feel competent will increase birthing women’s perceptions of receiving appropriate treatment. This was followed by a step-by-step implementation of different actions aiming to perform as few interventions as possible without compromising safety and maternal satisfaction. Among interventions, a 24-hour midwife coordinator function was instigated. A morning conference was implemented with daily teaching and mutual reflections over CSs the previous day. Coaching and supervision groups were made accessible to staff. A new “Birth planning clinic” addressed women’s childbirth fears and provided evaluation after obstetric emergencies (e.g., acute CS). New and optimised solutions were implemented for homestays for women with pregnancy-related problems as an alternative to in-hospital stays. For new parents, standard postpartum and post-surgical consultations were offered.

Clinical alterations included new training sessions to help prevent cesarean sections. This involved intrapartum ultrasound, manual rotation, rebozo for labor dystocia, and training for obstetricians to collaborate with midwives during normal birth. As a result, obstetricians were increasingly invited into the delivery rooms to be part of the decision-making team. Collaborative obstetric team training for emergencies was also part of these changes. Furthermore, to enhance transparency, close data monitoring of all births with visual monthly reports was introduced and evaluated regularly, and the statement about valuing vaginal birth was made public to women, their partners, and the local community. In total, 12 initiatives were implemented.

In S1 Supplementary the multi-component initiative is visualised and described in detail.

S1 SUPPLEMENTARY

This study aims to analyse the impact of a structured strategy to reduce CS rate implemented stepwise at HSJ from 2008 to 2017. The research question is if the intervention reduced the CS pattern without compromising foetal and maternal health. Further, if HSJ can show a reduction in the risk of CS, that is significantly different from similar hospitals.

# Material and Methods

The study uses prospectively collected data in a retrospective cohort design. For this purpose, data from the Danish Medical Birth Registry was sampled with additional Danish administrative registries to add further relevant patient data (e.g., education and citizenship). The dataset holds information on all Danish pregnancies from 22 gestational weeks and onwards, through childbirth until hospital discharge. In Denmark, all citizens hold a civil or temporary registration number. Thereby it is possible to register all contacts with healthcare providers [19]. Denmark has free access to hospitals during childbirth, which is why it is most likely that undocumented migrants giving birth are registered too [20]. Data are hosted in Statistics Denmark (MIPAC 705026). Data are based on routinely collected and aggregated data and handled according to European General Data Protection Regulation [21]. The study is approved by the Institutional Board at University College Copenhagen, approval ID: FK 2023/03.

**The population of interest:**

HSJ is a small/medium-sized Danish tertiary hospital located in an urban area of Denmark. The unit has six delivery rooms available and is staffed 24/7 by midwives, healthcare assistants and specialist obstetricians. Annually, the hospital has an approximate total of 1550 births. The labour ward has direct access to an intensive care unit and a neonatal intensive care unit (NICU).

We measured five years before the onset of the new strategy (2003-2007) and nine years after (2008-2017), including births from 1.January 2003 until 31. December 2017. During this period, a total of 938.365 pregnant people gave birth in Denmark. In this article, we use the term ‘women’ for all pregnant people. This choice is made for consistency within the study. Only births eligible for the strategy implemented at HSJ were included. Thereby preterm births <37+0 gestational weeks were excluded (N= 63,747), as well as births with no registered gestational age (N=17,518). The majority had low or no birth weight registered pointing to late abortions. To avoid confounding by indication, cases where CS was inevitable were excluded, such as placenta previa (N= 4310) and transverse/oblique foetal lie (N= 1612). Further, all pregnancies complicated by diabetes were excluded from the total sample, as the index hospital HSJ transfer diabetic women with insulin-dependent diabetes to a regional hospital with expertise (N=26,693). At HSJ, trial of labour after previous CS was restricted to two previous CS, which is why pregnancies with more than two previous CS were excluded (N=1076). The total population after restriction included 823,409 births in Denmark. Among these, 21,232 gave birth at HSJ. See S2 Supplementary for flowchart.

S2 SUPPLEMENTARY **The intervention of interest:**

The initiative evolved at HSJ from 2008 to 2017 to reduce the CS rate. As the strategy was implemented incrementally with a rollout in 2008, the analysis will be handled with 2008 as the year of introduction.

**The control group(s):**

This study will include a "single group" analysis (ITSA) and an analysis with a control group (CITSA). In the single-group analysis, the intervention period (2008-2017) in HSJ acts like HSJ's control group/counterfactual for the pre-intervention period (2003-2007). As a result, the trend in outcome in the preintervention period (e.g., CS) is compared to the trend in the intervention period. The assumption is that the population characteristics change slowly over time. Thereby the pre-intervention and the intervention population characteristics presumably are unaltered [22]. However, this approach cannot exclude confounding due to co-interventions or other events occurring around the time of the intervention (e.g., a national strategy for reducing CS rates). The latter problem can be handled by performing analyses similar to CITSA, which compares the development over time at HSJ with the development over time in the control groups [23]. When the index hospital is compared to the development over time at the control hospitals in the same time interval, it will be possible to observe whether time-dependent factors affect both populations [22–24]. To strengthen the analyses, two hypotheses were tested: First, if the trend over time before the intervention was initiated differed from the trend observed after the intervention began. The null hypothesis posited that the trend would remain unchanged. Secondly, the trend over time following the intervention at the index hospital differed from the trend among the control group. The null hypothesis suggested that the trend over time would be the same for both the index hospital and the controls.

The control group of hospitals that resemble HSJ was selected incrementally under the following criteria:

- The labour ward has existed throughout the years of interest; Twenty Danish hospitals existed from 2003-2017 (n=20).
- Approximately identical annual births as HSJ; Four units had a mean variation of births not exceeding +/- 500 annually compared to HSJ (n=4).
- Include the same level of care as HSJ: access to immediate operative support and neonatal intensive care (n=3).
- The control unit should not be in the same region to avoid the risk of local policies and initiatives (n=1). One hospital (Esbjerg) is excluded.

Two tertiary labour wards in urban areas met the inclusion criteria (Holbaek & Hjoerring). The control group was generated for robustness by merging these two units into one control group (N=46,417). A second group, including all other Danish hospitals except HSJ, mirrors Denmark's overall trends and policies over time (N=802,177) (S2 & S3 Supplementary).

**The outcomes of interest:**

In both single- and control-group analyses, the primary outcome is CS after implementing the stepwise initiative. CS is stratified into the timing of CS, that is “before onset of labour” or “intra partum”. Before onset of labour includes planned CS and acute CS before onset of labour. For intra partum CS, women must be in labour before a planned CS or CS caused by birth complications. Further, CS is stratified into parity (nulli- or multiparous). In the single group analysis, secondary outcomes where for the newborns: Apgar Score 7/ 5 minutes; admittance NICU >24 hours; and foetal death. For the mothers: instrumental birth (forceps or vacuum extraction), and uterine rupture (partial or total).

## Statistical analysis

Baseline characteristics for the groups were performed by combining the five years before implementing the strategy (2003-2007). Means and standard deviations (SD) are presented for continuous variables if normally distributed; otherwise, using median and interquartile range (1.-3. quartile). For dichotomous variables, percentages are used.

Baseline variables included parameters that may impact the risk of CS: mean maternal age, advanced maternal age (≥35 years), parity (nullipara, multipara), previous CS, level of education (low, medium, high), citizenship (Danish or not), severe preeclampsia (blood pressure ≥160 /110, proteinuria and/or indicators in blood tests or subjective symptoms), “medical diseases” relevant for pregnancy and childbirth registered by the general practitioner or obstetrician, mean Body Mass Index (BMI), pre-pregnancy obesity (BMI ≥30), smoking after 1. trimester, breech presentation, mean birthweight, birthweight ≥4000 gr, and foetal death (See Table 1).

Analyses were performed using single- and control group Interrupted Time Series Analysis (ITSA and CITSA). The year of childbirth served as the independent variable and was separated into half-years (n=30). The time was sequenced into a pre-intervention period (2003-2007; 10 observations) and a post-intervention period (2008-2017; 20 observations). The ITSA model fitted an ordinary least square line (OLS) pre-and post-intervention. We used Newey West to calculate standard errors and the Cumby-Huizinga test for autocorrelation [24]. If lags were present, the analyses were modified accordingly.

Visual descriptive figures present the difference between hospitals: annual CS rate (Fig.1); Outcomes are presented in Table 2 (ITSA) and Table 3 (CITSA), including the annual slopes (with a 95% Confidence interval) (95% CI) before and after the HSJ implementation. P-values present the statistical difference between the slopes pre- and postintervention for HSJ (Table 2) and the difference in slopes between HSJ and control in the post-intervention period (Table 3).

The potential impact of missing data depends largely on why these are missing data [25]. The current dataset can generate missing data randomly when a health care provider forgot to register a procedure or a condition. In such cases, the degree of missing data cannot be determined, but it may lead to underestimating events or conditions. However, economic refunds for healthcare activities incite the Danish hospitals to have optimal registration [19]. Other variables are mandatory to report (e.g., Apgar score). For these variables, the extent of missing registrations is measured. We included only variables where at least 95% were coded. This standard excluded valuable outcome variables such as postpartum haemorrhage, where coding before 2011 was inadequate. The variable with the highest frequency of missing data was BMI, with 10% missing. The lack of data was mainly due to an unsystematic registration in 2003. By restricting BMI data from 2004-2017 in the baseline tables, missing data on maternal BMI decreased to 3.8%. For NICU admittance, an organisational change at HSJ technically merged NICU admittance with the ordinary hospital stays for mother and baby from 2013 and onwards, which is why NICU cases cannot be separated. Thus, we restricted the post-intervention period regarding NICU to 2008-2012. Data on breastfeeding was available from an external health visitor dataset but included too much missing data to be relevant for the analyses. In Appendix 3 to 6 extra information on absolute numbers, parity, Robson classification and change in baseline characteristics over time is presented (see Results section)

STATA 17.0 (StataCorp 2021. Stata statistical software) was used for data management and analyses. All reported p-values are two-sided and with alpha=0.05 as the statistical level. For cohort reporting, the STROBE guidelines were used [26].

## Ethics Approval

## This study utilises retrospectively collected data hosted by Statistics Denmark. All data were fully anonymised before being made available to the researchers. The data are publicly presented in an aggregated format, not requiring patient consent. The dataset used for the analysis is hosted by Statistics Denmark (project ID: MIPAC 705026) and is administered by University College Copenhagen. The Institutional Review Board at University College Copenhagen approved the study (approval no. FK 2023/03).

## Patient and Public Involvement

# To enhance public involvement, we contacted the Danish NGO "Parenthood and Childbirth", a democratic member-driven organisation working to promote women's influence on pregnancy and childbirth. The group was involved before any protocol was outlined and outcome measures were developed in collaboration. Some outcomes were impossible to monitor according to the variables present in the dataset (e.g., length of breastfeeding, maternal satisfaction, and postpartum haemorrhage). Dissemination of the study entailed "Parenthood and Childbirth" using their socially available platforms to promote the study and to discuss the results.

**Results**

After exclusion, the total eligible population was n=823,456. Of those, 21,232 gave birth at HSJ, whereas 46,417 gave birth at the two control hospitals. The Danish CS trend includes all except HSJ, which is n=802,177 (S2 Supplementary ).

**Descriptive results:**

**Baseline characteristics:**

Table 1 shows baseline characteristics for the index population (HSJ) compared to controls at baseline. We used the pre-intervention period (2003-2007) to assess baseline.

**Table 1**: Baseline characteristics for HSJ, control and Denmark (without HSJ) in the preintervention period, N= 289.045.

| **Baseline characteristics, pre-intervention period, 2003-2007** | | | |
| --- | --- | --- | --- |
| ***Characteristic*** | **HSJ**  n=7.129 | **Control**  n=19.215 | **Denmark**  n=281.916 |
| **Age, mean ±SD** | 29.5 ±4.9 | 29.3 ±4.9 | 30.1 ±4.7 |
| **Age, 35+, n (%)** | 1075 (15.1) | 2786 (14.5) | 49,754 (17.7) |
| **Nulliparous, n (%)** | 4330 (60.7) | 11,376 (63.9) | 157,167 (56.5) |
| **Multiparous, n (%)** | 2799 (39.3) | 6435 (36.1) | 121,221 (43.5) |
| **Previous caesarean, n (%)** | 768 (17.4) | 1828 (16.1) | 26,182 (16.7) |
| **Educational level, n (%) ^a^** |  |  |  |
| Low | 1019 (14.9) | 2758 (14.9) | 32,783 (12.2) |
| Medium | 3407 (49.9) | 9437 (51.0) | 117,267 (43.7) |
| High | 2422 (35.4) | 6316 (34.1) | 118,533 (44.1) |
| **Not Danish Citizenship, n (%)** | 797 (11.3) | 1811 (9.5) | 35,935 (12.9) |
| **Severe preeclampsia, n (%)** | 12 (0.17) | 66 (0.34) | 839 (0.30) |
| **Medical diseases_,_ n (%)^b^** | 21 (0.29) | 421 (2.2) | 5074 (1.8) |
| **BMI, median (1.-3. quartile) ^c^** | 23.9 (21.5-27.6) | 23.8 (21.3-27.4) | 23.0 (20.8-26.1) |
| **Obese, BMI>30, n (%) ^c^** | 766 (15.0) | 2047 (13.8) | 22,282 (10.6) |
| **Smoking after 1. trimester, n (%)** | 1316 (18.6) | 3966 (20.9) | 39,835 (14.4) |
| **Breech presentation, n (%)** | 305 (4.3) | 766 (4.0) | 11,531 (4.11) |
| **Birthweight gr., mean ±SD** | 3531 ±521 | 3543 ±505 | 3549 ±498 |
| **Birthweight >4000 gr, n (%)** | 1254 (19.1) | 3282 (18.4) | 48,327 (18.4) |
| **Foetal death, n (%) ^d^** | 22 (0.31) | 32 (0.17) | 509 (0.18) |
| a. Educational level: Low=basic education; Middel= secondary education/ skilled worker/short higher education; High=medium higher/bachelor/long higher/ph.d | | | |
| b. Medical disease that may impact pregnancy and childbirth. The general practitioner or obstetrician scored them.  c. only data from 2004-2007. HSJ n=5701 / control n=15.708 / Denmark n=210.889  d. In available data, prelabour and intrapartum deaths cannot be distinguished. However, in Denmark, almost all deaths happen before the onset of labour. | | | |

HSJ and control differ marginally. At HSJ, the population has 3.2% fewer nulliparous and 1.3% more with a previous CS, 1,8% more are not Danish, 1,9% less are diagnosed with medical diseases, and 1.2% more have a BMI >30. There is a difference in foetal death at baseline (0.31% vs. 0.14%). HSJ and control represent urban and smaller Danish labour wards heterogeneous to the overall Danish characteristics. At baseline, the general Danish population include more multiparous, more aged >35, more women with higher education, fewer with obesity and fewer smokers than the HSJ and the control population (see Table 1).

**Total CS during 2003-2017**

Figure 1 shows the overall CS rate at HSJ, the control hospitals and Denmark (excluded HSJ).

**Fig. 1**, Caesarean section rate 2003-2017 among HSJ, control and Denmark.

Presented in percent (%) with 95% CI. Dotted line: time for initiation of intervention.

FIGURE 1

Denmark had a stable CS rate at approximately 18% over the timespan, but in the preintervention period between 2007-2013, an increasing trend from 18% to 20% appeared. The CS rate rose at both HSJ and the control hospitals during this period. After 2008, the control group increased their CS rate to approximately 22-23%, whereas HSJ decreased to 12% annually (2017). For absolute numbers and percent, see

S3 SUPPLEMENTARY. Furthermore, S4 SUPPLEMENTARY stratify CS rates by parity and timing (elective vs. acute). As extra information, S5 SUPPLEMENTARY presents CS into the Ten Group Classifications System [3] and S6 SUPPLEMENTARY shows changes in maternal characteristics over 2003-2017 among HSJ and control.

**Time Series Analysis:**

**Single analyses: HSJ pre-period versus the intervention period**

Single-group analyses statistically calculate if the trend at HSJ differs between the pre- and the intervention period. Table 2 presents outcomes for the single-group ITSA with a pre-intervention trend, an intervention trend and a significance test to determine if the difference in trends is significant (see Table 2).

**Table 2.** Timeseries analyses HSJ trend before- and during intervention

|  | **Pre-intervention trend** | **Trend during intervention** | **Difference in trends** |
| --- | --- | --- | --- |
| **Maternal outcome** | % per year (95% CI) | % per year (95% CI) | P-value |
| Total caesarean | 0.20 (-0.36 to 0.77) | -0.87 (-1.16 to -0.56) | 0.002 |
| Total caesarean, nullipara | 0.28 (- 0.29 to 0.85) | -0.35 ( -0.51 to -0.19) | 0.04 |
| Total caesarean, multipara | -0.08 (- 0.41 to 0.25) | -0.51 ( -0.71 to -0.31) | 0.03 |
| Caesarean elective or subacute | 0.09 (-0.42 to 0.60) | -0.60 (-0.77 to -0.43) | 0.02 |
| Caesarean intra partum | 0.12 (-0.22 to 0.47) | -0.27 (-0.49- to -0.05) | 0.07 |
| Uterine rupture | 0.013 (-0.046 to 0.049) | 0.012 (-0.013 to 0.015) | 1.00 |
| Instrumental birth | -0.27 (-0.76 to 0.22) | -0.22 (-0.46 to 0.00) | 0.06 |
| **Perinatal outcome** |  |  |  |
| Apgar score < 7/ 5 minutes | 0.17 (0.07 to 0.26) | 0.02 (-0.04 to 0.08) | 0.009 |
| NICU> 24 hours* | 0.40 (0.002 to 0.79) | 0.24 (-0.30 to 0.79) | 0.43 |
| Foetal death | -0.08 (-0.15 to 0.005) | -0.02 (-0.61 to 0.004) | 0.15 |
| * NICU is only measured until 2012 inclusive, as an organisational merging of units makes it impossible to distinguish NICU cases in dataset | | | |

The CS rates increased in the preintervention period for total CS (for example, +0.20% annually), nulliparous, and elective/subacute CS. After introducing the strategy, these groups have a statistically significant decrease in CS rates, which reflects a negative trend (e.g., overall CS rate decreased post-intervention by -0.87% annually, (p=0.002)). Of other significant findings, multiparous had a slightly decreasing trend in the pre-intervention period, but this trend decreased more rapidly after the HSJ intervention in 2008 (p=0.03).

For the secondary outcomes, only Apgar <7/5 minutes changed significantly. After an upgoing trend pre-intervention (+0.17% annually) Apgar <7/5 had a steady state post-intervention (+0.02%) (p=0.009). Uterine rupture, instrumental delivery, admittance to NICU and risk of foetal death remained unaltered.

**Multiple analyses: HSJ versus control group**

In the CITSA, we tested if national trends regarding CS rates differed from HSJ and the control group. Figure 2 shows CITSA with a pre-intervention trend and a post-intervention trend.

**Fig.2.** Caesarean section. HSJ versus control hospitals, CITSA

FIGURE 2

Dots: biannual CS rates at HSJ (red) and control (white). Ordinary least square line (OLS) for each hospital before and after the intervention. The dotted line (2008) represents the time of implementation.

Table 3 presents trends at HSJ and control in the post-intervention period and a significance test to determine the difference. See S4 Supplementary for visual inspection.

**Table 3.** Time series comparing trends at HSJ and control hospitals in the pre-intervention and intervention period. Significance test determine if trends differ between groups.

|  | **Difference between groups** | | | | |
| --- | --- | --- | --- | --- | --- |
|  | **Pre-intervention** | **Post difference between groups** | | **Post-intervention trend** | |
| **Maternal outcome** | P-value | **HSJ** % per year (95% CI) | **Control hospitals** % per year (95% CI) | **Difference HSJ/control**  % per year (95% CI) | P-value |
| Total Caesarean | 0.10 | -0.87 (-1.16 to -0.56) | -0.12 (-0.29 to 0.06) | -0.75 (-1.1 to -0.41) | <0.001 |
| Total caesarean, nullipara | 0.02 | -0.35 ( -0.51 to -0.19) | 0.53 (0.42 to 0.63) | -0.87 (-1.06 to -0.69) | <0.001 |
| Total caesarean, multipara | 0.00 | -0.51 ( -0.71 to -0.31) | -0.26 (-0.55 to 0.02) | -0.25 (-0.59 to 0.1) | 0.15 |
| Caesarean before onset of labour | 0.21 | -0.60 (-0.77 to -0.43) | 0.04 (-0.10 to 0.19) | -0.64 (-0.86 to -0.42) | <0.001 |
| Caesarean intra partum | 0.45 | -0.27 (-0.49- to -0.05) | -0.16 (-0.26 to -0.06) | 0.11 (-0.34 to 0.12) | 0.34 |

The overall CS trend did not differ in the pre-intervention period (p=0.10). In the post-intervention period, an annual decrease in CS rates is noted at HSJ (-0.87 % annually) and the control group ( -0.12% annually) (p<0.001). The overall decrease points to a Danish awareness of stabilizing or reducing the CS rates, as HSJ and control decreased after 2008. Both HSJ and control managed to change CS increase from the pre-intervention period to the post-intervention period, but HSJ with an extra -0.75% annually (p<0.001). Stratified by parity, nullipara has different trends after the intervention. HSJ reduce the risk of CS by -0.35% annually, whereas there is an increase in the control hospitals by +0.53% annually. The difference is significant (p<0.001).

Looking at CS before the onset of labour, HSJ has an annual reduction in the post-trend period of -0.60 % annually, whereas the control has a non-significant increase of 0.04% annually. With this, HSJ reduced the CS before the onset of labour by -0.64% annually compared to the control group (p<0.000).

# Discussion

## Principal findings

Data analysis showed a statistically significant change in trend and a progressive reduction in CS rates at HSJ as the initiative was implemented (2008-2017). The CS rate decreased at HSJ from 21.1% (95% CI [19.0-23.5]) in 2008 to 12.0% (95% CI [10.4-13.6]) in 2017. This decline corresponds to a mean decrease of 0.87% annually (p=0.002) and a relative 43% decrease over the 9-years. Using a control group, we found a national trend towards reducing CS rates. However, the decrease in CS rate in the control group was modest and insignificant (-0.12% annually). Compared to the control group, HSJ's implementation provided an extra 0.75% annual reduction in CS rates (p<0.001). We found no negative consequences of the initiative on instrumental birth, uterine ruptures, NICU>24 hours or foetal death.

## Strengths and limitations of this study

Similar studies monitoring an intervention to reduce CS rates are often conducted as pre- and post-interventions studies without a control group and without accounting for the underlying trend [27,28] or as randomised designs running over a short period [29]. This study's strength is in comparing the use of ITSA with a control group (CITSA) as this design provides a robust quasi-experimental alternative to the randomised design [30]. Moreover, by using multiple pre- and post-intervention observations, we account for the underlying trends. As the characteristics of the population tend to change slowly over time, confounding factors are rarely a problem [23,24]. Further, using a control group strengthens the results by excluding confounding due to time-dependent factors affecting HSJ and controls [22–24]. Also, despite the retrospective design, data were prospectively collected from health personnel unaware of the purpose of the study.

A limitation of the study is the absence of a few relevant variables. HSJ provides merged and thus inappropriate data for NICU>24 hours from 2013. Further, our collaborating NGO organisation requested data on the length of breastfeeding and maternal satisfaction. Both measures were either inadequately collected or absent in the Danish registers. Postpartum haemorrhage (PPH) was not routinely collected before 2011, and thus not possible to monitor before and after the intervention. However, on publicly available health data, HSJ had a lower risk of PPH>1000 ml. than the national average (2016: 5,7% vs. 6,7%; 2017: 5,6% vs. 7,0%)[31]. Compared to other studies [28,29], another limitation is that the changes at HSJ were conducted incrementally and not under any formal randomised design. Finally, although the results suggest a causal relationship between the overall HSJ initiative and the CS rate at the labour ward, we cannot assess the effectiveness of the individual components of this initiative. Stakeholders’ attitudes and experiences are essential when interpreting and explaining complex interventions' effectiveness results [32]. A qualitative process evaluation of the initiative could have added valuable information on the compliance with and acceptability of the intervention components among the obstetric staff and women.

**The present study in comparison to previous studies**

A cluster randomised trial at 32 hospitals in Quebec, has assessed the impact of audits on CS, providing feedback, and implementing best practices to reduce CS rates [29]. After the implementation of this initiative, the CS rates were significantly lowered, yet only by 0.7% in total. The difference between Quebec and HSJ may be in the organisational structure of the implementation. The Quebec initiative was handled by a selected audit committee performing four annual feedbacks and education of the health team. The HSJ initiative had an organisational bottom-up approach, as the initiative to decrease the CS rate was brought forward by midwives and obstetricians at the unit and then supported by the obstetric management.

Lanham and colleagues argue that the degree of connectedness or interrelatedness among project participants impacts the uptake of an intervention [33]. As documented in other studies [4,34], components aiming to enhance interdisciplinary cooperation in the HSJ initiative may have functioned as important facilitators for the initiatives’ implementation. A systematic qualitative assessment of promoters for reducing CS rates has shown that professional teamwork between healthcare providers is pivotal for tackling unnecessary CSs [35]. The highest CS rates were among institutions reporting challenges in achieving collaborative work between midwives and obstetricians and non-collaboration in the organisational culture and policy documents [35]. These results align with the HSJ initiative, where collaborative teamwork between obstetricians and midwives was promoted through clinical skills training, supervision, mutual daily obstetric conferences, and obstetricians attending normal births (S1 Supplementary).

Another study has investigated a multicomponent intervention restricting stakeholders' and private obstetricians’ access to order a CS and implementing a senior clinical management team to approve elective CS [28]. Results showed the CS rate declined from 33.7% to 22.4%. The main contribution was a reduction in elective CS for non-obstetric reasons, which indicates how the organisation of birth care and cultural attitudes may impact the overall CS rate. The endpoint at 22.4% is higher than the initial CS rate at HSJ before the initiative, suggesting cultural differences between the two initiatives. As previously documented, stakeholders’ attitudes and beliefs towards CS may impact CS intervention rates [35,36]*.* Indeed, a recent review found clinicians’ personal beliefs to be a critical factor in the decision to perform a CS [37]. Personal beliefs regarded perceptions of risks associated with vaginal birth and personal preferences. These were influenced by professional agreement and disagreement among obstetricians and midwives [37].

Finally, a 9-component organisational and cultural change in obstetric care, aimed at Robson 1; nulliparous, has been investigated for its effect [27]. Results showed a similar reduction in CS as HSJ, from 20% to 11% during nine years. As at HSJ, the hospital's CS rate has remained low at 9% (2022) [38]. What unites the two initiatives is the continuous emphasis on the common belief in safe vaginal births, the inter-disciplinary teamwork, skills training, and the daily conference to discuss cases of CS. Differences between the two initiatives include HSJ's focus on all parities and additional components directed towards subsequent pregnancies.

**Implications for practice and future research**

Our findings show that multicomponent initiatives can reduce cesarian section rates without compromising maternal or foetal health. Thus, investing in such initiatives may be worth the effort. One possible explanation for the initiative’s success is the HSJ leadership. As argued by Braithwaite and colleagues, hands-on approaches by leaders play an essential role in adopting innovations [39]. Also, sufficient time is required to create adequate information flow and feedback and observe an intervention's impact [40]. The fact that the twelve components of the HSJ intervention were implemented over nine years may allow the necessary time for such processes to take place. As demonstrated in Figure 1, the CS rate at HSJ decreased throughout the implementation period, suggesting that components implemented early and late contributed to this decrease in CS.

Five years after the HSJ finished the implementation of the initiative, the CS rate is still low (13% in 2022) [17], suggesting a high sustainability of the initiative. Kingdon et al. argue that for practice changes to be persistent, key stakeholders must be involved in both the development and implementation of an intervention, as resistance often originates from a feeling of exclusion resulting in a lack of ownership [35]. In the HSJ initiative, there has been interdisciplinary involvement in both the initiative's development and implementation phases [41]. Finally, so far most qualitative studies on CS interventions have focused on maternity care provider perspectives[16,36]. While women’s perspectives on CS, e.g., regarding inclusion in decision-making processes, giving informed consent, and fear of vaginal childbirth, are well examined [16,35,42], the evidence base on how women respond to different CS intervention components is currently limited [4]. Future research is needed on the acceptability and satisfaction of such interventions among users. In the current study, such outcomes were not available. However, a national satisfaction survey in 2017 showed that women at HSJ generally scored more satisfied with antenatal care and had similar scores as the national average regarding overall satisfaction with birth [43].

# Conclusion

This study shows the possible impact of a structured cross-sectional effort to reduce caesarean section at a Danish hospital. During nine years, a relative 43% decrease in caesarean section rate was accomplished without compromising the health of the mother or the newborn. The intervention demonstrates the feasibility and effectiveness of this interdisciplinary structured change in culture and clinical practice. If this complex intervention can be implemented elsewhere, it may have a huge potential positive impact on maternal and foetal health.

*Wordcount 4724*

## Author Contributions

ER and HJ planned the study. ER and OO analysed data. ER, KGN, OO, ALH & HJ wrote the manuscript in close collaboration. All authors revised the manuscript and accepted the final version. ER is the guarantor.

## Acknowledgement

We are grateful for the help provided by the HSJ hospital regarding data and historical knowledge. Further, we acknowledge the fruitful collaboration with the “Parenthood and Childbirth” organisation in the initial planning of the study.

**Competing interests**

ER, KGN, OO, ALH and HJ have nothing to disclose. The study had no external financial support and was funded by University College Copenhagen as part of ER and HJ´s employment and by The Research Unit for General Practice, Department of Public Health, University of Copenhagen as part of OO´s employment.

**Funding**

This research received no specific grant from any funding agency in the public, commercial or not-for-profit sectors.

**Data sharing statement**

All data relevant to the study are included in the article or uploaded as supplementary information.

**References**

[1]	Betran AP, Ye J, Moller A-B, Souza JP, Zhang J. Trends and projections of caesarean section rates: global and regional estimates. BMJ Glob Health 2021;6. https://doi.org/10.1136/bmjgh-2021-005671.

[2]	Betran AP, Torloni MR, Zhang J, Ye J, Mikolajczyk R, Deneux-Tharaux C, et al. What is the optimal rate of caesarean section at population level? A systematic review of ecologic studies. Reprod Health 2015; 12:56–7. https://doi.org/10.1186/s12978-015-0043-6 [doi].

[3]	World Health Organization. WHO Statement on Caesarean Section Rates. World Health Organization, Geneva, Switzerland: Department of Reproductive Health and Research; 2015.

[4]	World Health Organization. Non-clinical interventions to reduce unnecessary cesarean sections. Geneva: 2018.

[5]	Health at a Glance 2019. OECD; 2019. https://doi.org/10.1787/4dd50c09-en.

[6]	Souza JP, Gülmezoglu A, Lumbiganon P, Laopaiboon M, Carroli G, Fawole B, et al. Caesarean section without medical indications is associated with an increased  risk of adverse short-term maternal outcomes: the 2004-2008 WHO Global Survey on Maternal and Perinatal Health. BMC Med 2010; 8:71. https://doi.org/10.1186/1741-7015-8-71.

[7]	Keag OE, Norman JE, Stock SJ. Long-term risks and benefits associated with cesarean delivery for mother, baby, and subsequent pregnancies: Systematic review and meta-analysis. PLoS Med 2018;15: e1002494. https://doi.org/10.1371/journal.pmed.1002494.

[8]	Timor-Tritsch IE, Monteagudo A. Unforeseen consequences of the increasing rate of cesarean deliveries: early placenta accreta and cesarean scar pregnancy. A review. Am J Obstet Gynecol 2012; 207:14–29. https://doi.org/10.1016/j.ajog.2012.03.007.

[9]	Cook JR, Jarvis S, Knight M, Dhanjal MK. Multiple repeat caesarean section in the UK: incidence and consequences to mother and child. A national, prospective, cohort study. BJOG 2013; 120:85–91. https://doi.org/10.1111/1471-0528.12010.

[10]	Marshall NE, Fu R, Guise J-M. Impact of multiple cesarean deliveries on maternal morbidity: a systematic review. Am J Obstet Gynecol 2011; 205:262.e1-8. https://doi.org/10.1016/j.ajog.2011.06.035.

[11]	Sandall J, Tribe RM, Avery L, Mola G, Visser GH, Homer CS, et al. Short-term and long-term effects of caesarean section on the health of women and children. The Lancet 2018; 392:1349–57. https://doi.org/10.1016/S0140-6736(18)31930-5.

[12]	Lobel M, DeLuca RS. Psychosocial sequelae of cesarean delivery: review and analysis of their causes and implications. Soc Sci Med 2007; 64:2272–84. https://doi.org/10.1016/j.socscimed.2007.02.028.

[13]	Betrán AP, Temmerman M, Kingdon C, Mohiddin A, Opiyo N, Torloni MR, et al. Interventions to reduce unnecessary caesarean sections in healthy women and babies. Lancet 2018; 392:1358–68. https://doi.org/10.1016/S0140-6736(18)31927-5.

[14]	Caughey AB, Cahill AG, Guise J-M, Rouse DJ. Safe prevention of the primary cesarean delivery. Am J Obstet Gynecol 2014; 210:179–93. https://doi.org/10.1016/j.ajog.2014.01.026.

[15]	Osterman M. Changes in Primary and Repeat Cesarean Delivery : 2022.

[16]	Chen I, Opiyo N, Tavender E, Mortazhejri S, Rader T, Petkovic J, et al. Non-clinical interventions for reducing unnecessary caesarean section. Cochrane Database Syst Rev 2018;9:CD005528. https://doi.org/10.1002/14651858.CD005528.pub3.

[17]	Danish Health Data Board. Fødselsregisteret [Danish National Birth Register] 2023. https://www.esundhed.dk/home/emner/graviditet foedsler og boern/nyfoedte og foedsler 1997-.

[18]	Waldenström U, Nilsson CA. Women’s satisfaction with birth center care: a randomized, controlled study. Birth 1993; 20:3–13. https://doi.org/10.1111/j.1523-536x.1993.tb00173.x.

[19]	Bliddal M, Broe A, Pottegard A, Olsen J, Langhoff-Roos J. The Danish Medical Birth Register. Eur J Epidemiol 2018; 33:27–36. https://doi.org/10.1007/s10654-018-0356-1 [doi].

[20]	Funge JK, Boye MC, Johnsen H, Nørredam M. “No Papers. No Doctor”: A Qualitative Study of Access to Maternity Care Services for Undocumented Immigrant Women in Denmark. Int J Environ Res Public Health 2020;17:1–14. https://doi.org/10.3390/ijerph17186503.

[21]	European Commission. General Data Protection Regulation. EUR-Lex; 2018.

[22]	Lopez Bernal J, Soumerai S, Gasparrini A. A methodological framework for model selection in interrupted time series studies. J Clin Epidemiol 2018; 103:82–91. https://doi.org/10.1016/j.jclinepi.2018.05.026.

[23]	Bernal JL, Cummins S, Gasparrini A. The use of controls in interrupted time series studies of public health interventions. Int J Epidemiol 2018; 47:2082–93. https://doi.org/10.1093/ije/dyy135.

[24]	Linden A. Conducting Interrupted Time-series Analysis for Single- and Multiple-group Conducting interrupted time-series analysis for single- and multiple-group comparisons 2015. https://doi.org/10.1177/1536867X1501500208.

[25]	Sainani KL. Dealing With Missing Data. PM R 2015; 7:990–4. https://doi.org/10.1016/j.pmrj.2015.07.011.

[26]	Vandenbroucke JP, Elm E Von, Altman DG, Gøtzsche PC, Mulrow CD, Pocock SJ, et al. Annals of Internal Medicine Academia and Clinic Strengthening the Reporting of Observational Studies in Epidemiology ( STROBE ): Explanation and Elaboration. Ann Intern Med 2007; 147:163–94.

[27]	Blomberg M. Avoiding the first cesarean section--results of structured organizational and cultural changes. Acta Obstet Gynecol Scand 2016;95:580–6. https://doi.org/10.1111/aogs.12872.

[28]	Zahumensky J, Psenkova P, Dolezal P, Otapkova P, Papcun P, Ferianec V, et al. Impact of implementing a multifaceted intervention to reduce rates of cesarean section: A quality-improvement study. Int J Gynaecol Obstet 2020;151:244–8. https://doi.org/10.1002/ijgo.13345.

[29]	Chaillet N, Dumont A, Abrahamowicz M, Pasquier J-C, Audibert F, Monnier P, et al. A cluster-randomized trial to reduce cesarean delivery rates in Quebec. N Engl J Med 2015; 372:1710–21. https://doi.org/10.1056/NEJMoa1407120.

[30]	Kontopantelis E, Doran T, Springate DA, Buchan I, Reeves D. Regression based quasi-experimental approach when randomisation is not an option: Interrupted time series analysis. BMJ (Online) 2015; 350:1–4. https://doi.org/10.1136/bmj.h2750.

[31]	ESundhed. Nyfødte og fødsler (1997-). The Danish Health Data Authority n.d. https://www.esundhed.dk/home/emner/graviditet foedsler og boern/nyfoedte og foedsler 1997-.

[32]	Skivington K, Matthews L, Simpson SA, Craig P, Baird J, Blazeby JM, et al. A new framework for developing and evaluating complex interventions: update of Medical Research Council guidance. BMJ 2021;374: n2061. https://doi.org/10.1136/bmj.n2061.

[33]	Lanham HJ, Leykum LK, Taylor BS, McCannon CJ, Lindberg C, Lester RT. How complexity science can inform scale-up and spread in health care:  understanding the role of self-organization in variation across local contexts. Soc Sci Med 2013; 93:194–202. https://doi.org/10.1016/j.socscimed.2012.05.040.

[34]	Marshall JL, Spiby H, McCormick F. Evaluating the “Focus on Normal Birth and Reducing Caesarean section Rates Rapid Improvement Programme”: A mixed method study in England. Midwifery 2015; 31:332–40. https://doi.org/10.1016/j.midw.2014.10.005.

[35]	Kingdon C, Downe S, Betran AP. Non-clinical interventions to reduce unnecessary caesarean section targeted at organisations, facilities and systems: Systematic review of qualitative studies. PLoS One 2018;13:e0203274. https://doi.org/10.1371/journal.pone.0203274.

[36]	Betrán AP, Temmerman M, Kingdon C, Mohiddin A, Opiyo N, Torloni MR, et al. Interventions to reduce unnecessary caesarean sections in healthy women and babies. Lancet 2018; 392:1358–68. https://doi.org/10.1016/S0140-6736(18)31927-5.

[37]	Panda S, Begley C, Daly D. Correction: Clinicians’ views of factors influencing decision-making for caesarean section: A systematic review and metasynthesis of qualitative, quantitative and mixed methods studies. PLoS One 2018;13:e0202688. https://doi.org/10.1371/journal.pone.0202688.

[38]	Socialstyrelsen. Sveriges officielle statistik ; Graviditeter, förlossningar och nyfödda barn [Sweden’s Official Statistics; Pregnancies, Deliveries, and Newborn]. Sverige: Socialstyrelsen; 2023.

[39]	Braithwaite J, Churruca K, Long JC, Ellis LA, Herkes J. When complexity science meets implementation science: a theoretical and empirical analysis of systems change. BMC Med 2018; 16:63. https://doi.org/10.1186/s12916-018-1057-z.

[40]	Moore GF, Evans RE, Hawkins J, Littlecott H, Melendez-Torres GJ, Bonell C, et al. From complex social interventions to interventions in complex social systems:  Future directions and unresolved questions for intervention development and evaluation. Evaluation (Lond) 2019; 25:23–45. https://doi.org/10.1177/1356389018803219.

[41]	Funch M. I Aabenraa er jordemødrene glade [In Aabenraa, the midwives are pleased]. Kristligt Dagblad 2022:1.

[42]	Declercq ER, Sakala C, Corry MP, Applebaum S, Herrlich A. Major Survey Findings of Listening to Mothers (SM) III: Pregnancy and Birth:  Report of the Third National U.S. Survey of Women’s Childbearing Experiences. J Perinat Educ 2014; 23:9–16. https://doi.org/10.1891/1058-1243.23.1.9.

[43]	Den Landsdækkende Undersøgelse af Patientoplevelser 2017, fødende [National Survey of Patient Experiences, Maternity Care 2017]Accessed Nov.2024 at: [Landsdækkende Undersøgelse af Patientoplevelser blandt Fødende 2017 (LUP Fødende 2017)](https://www.regionh.dk/patientinddragelse/udgivelser/udgivelser/Sider/LUP-F%C3%B8dende-2017.aspx)
